# Impact of investigator initiated trials and industry sponsored trials on medical practice (IMPACT): rationale and study design

**DOI:** 10.1186/s12874-020-01125-5

**Published:** 2020-10-02

**Authors:** E. Nury, K. Bischoff, K. Wollmann, K. Nitschke, S. Lohner, M. Schumacher, G. Rücker, A. Blümle

**Affiliations:** 1 Institute for Evidence in Medicine (for Cochrane Germany Foundation), Faculty of Medicine and Medical Center, University of Freiburg, Breisacher Str. 86, 79110 Freiburg, Germany; 2 Cochrane Hungary, Clinical Centre of the University of Pécs, Medical School, University of Pécs, Rákóczi út 2, Pécs, 7623 Hungary; 3 Institute of Medical Biometry and Statistics, Faculty of Medicine and Medical Center, University of Freiburg, Stefan-Meier-Straße 26, 79104 Freiburg, Germany; 4 Clinical Trials Unit, Faculty of Medicine and Medical Center, University of Freiburg, Elsässer Straße 2, 79110 Freiburg, Germany

**Keywords:** Randomized controlled trial, Study registry, Access to information, Evidence-based medicine, Systematic reviews, Clinical guidelines, Knowledge translation, Clinical decision making, Investigator initiated trials, Industry sponsored trials

## Abstract

**Background:**

The German Research Foundation (DFG) and the Federal Ministry of Education and Research (BMBF) initiated large research programs to foster high quality clinical research in the academic area. These investigator initiated trials (IITs) cover important areas of medical research and often go beyond the scope of industry sponsored trials (ISTs). The purpose of this project was to understand to what extent results of randomized controlled IITs and ISTs have an impact on medical practice, measured by their availability for decisions in healthcare and their implementation in clinical practice. We aimed to determine study characteristics influencing a trial’s impact such as type of sponsor and place of conduct. In this article, we describe the rationale and design of this project and present the characteristics of the trials included in our study cohort.

**Methods:**

The research impact of the following sub-cohorts was compared: German IITs (funded by DFG and BMBF or by other German non-commercial organizations), international IITs (without German contribution), German ISTs, and international ISTs. Trials included were drawn from the DFG−/BMBF-Websites, the German Clinical Trials Register, and from ClinicalTrials.gov. Research impact was measured as follows: 1) proportion of published trials, 2) time to publication, 3) proportion of publications appropriately indexed in biomedical databases, 4) proportion of openly accessible publications, 5) broadness of publication’s target group, 6) citation of publications by systematic reviews or meta-analyses, and 7) appearance of publications or citing systematic reviews or meta-analyses in clinical practice guidelines. We also aimed to identify study characteristics associated with the impact of trials.

**Results:**

We included 691 trials: 120 German IITs, 200 International IITs, 171 German ISTs and 200 International ISTs. The median number of participants was 150, 30% were international trials and 70% national trials, 48% drug-trials and 52% non-drug trials. Overall, 72% of the trials had one pre-defined primary endpoint, 28% two or more (max. 36).

**Conclusions:**

The results of this project deepen our understanding of the impact of biomedical research on clinical practice and healthcare policy, add important insights for the efficient allocation of scarce research resources and may facilitate providing accountability to the different stakeholders involved.

## Background

Evidence-based decisions in health-care should be based on the best available research results generated in clinical trials. Therefore it is important that all research findings are reported transparently and made publicly available so that they can be used in medical practice to ensure an appropriate and up-to-date treatment of individual patients [1]. Consequently, it is crucial that results from all clinical studies are fully published, that the publications are included and findable e. g. in biomedical databases, and that the articles are accessible. Previous publications report that only about half of clinical study findings are published as full-text article in peer-reviewed journals [2, 3]. This implies that a large body of informative evidence generated in clinical studies is lost and that secondary research articles such as systematic reviews or meta-analyses and clinical guidelines are built on a limited and possibly biased dataset. In the worst case this results in biased estimates of treatment effects [4]. This in turn can lead to a wrong medical decision that may ultimately result in a non-optimal treatment of patients [5]. Several studies showed that the effect estimate of a study outcome can change when also including unpublished study results in the meta-analyses. In these cases experimental treatments may prove to be more harmful and no more efficacious than the comparison treatment, e. g. standard treatment or placebo [5–7].

Several trial characteristics, such as a large sample size as well as a large number and internationality of participating study sites have been shown to be associated with a higher publication rate [3]. Also positive results (favoring the experimental treatment) and statistically significant results are published significantly more often and sooner than negative results (those favoring the control treatment) and statistically non-significant results [8–11]. A majority of articles indicate that industry sponsored trials (ISTs) might be more susceptible to this so-called reporting bias [12–21], but there are also some findings [22] indicating that non-publication is an issue in investigator initiated trials (IITs) as well. A Health Technology Assessment report by Song et al. on the dissemination and publication of research findings found that the main reasons stated by academic investigators for not publishing their studies consisted of ‘a lack-of-time or low priority’, followed by ‘results not important enough’ and ‘journal rejection’ [23]. These results are in line with a systematic review on the reasons provided by authors of conference abstracts for not publishing results as full articles [24]. Prospective trial registration may effectively address the issue of non-publication and has become an important measure to reveal studies that remained unpublished [25–27]. The Lancet highlighted this important issue in a five-article-series [28–33], summarizing the concerns and giving recommendations on how to increase value and reduce waste in biomedical research, as well as proposing metrics for stakeholders to monitor the implementation of the recommendations.

It is evident that under-reporting thwarts knowledge translation from research into practice [8]. Indicators for whether or not knowledge translation has been successful are the use of research findings in subsequent research and their implementation in healthcare. Sarli and colleagues [34] developed a framework (Becker Medical Library Model for Assessment of Research Impact) in which they distinguished four concepts to assess the impact of a study, 1) research output, i.e. the products generated or disseminated from the research study, e. g. publication of study results; 2) knowledge transfer, the awareness and use of research outputs created or disseminated by a research study, e. g. the study is cited in a journal article or systematic review; 3) clinical implementation, i.e. the application or adoption of research outputs in clinical practice, e. g. measured by citation in clinical or practice guidelines; and 4) community benefit, i.e. the enhancement of both community health outcomes, e. g. clinical well-being of community members, and cost-effectiveness of disease management and treatment.

This project covers the first three concepts, with a special focus on clinical implementation and research outputs originating from IITs, which in our project comprise clinical trials that were initiated at academic institutions and funded non-commercially, compared to commercially initiated and funded ISTs. IITs and ISTs usually play different roles within healthcare research [35]. Whereas IITs typically have no commercial interest and focus on issues important to patients and society as well as on (healthcare) knowledge expansion [36], ISTs focus on the commercial translation of research into clinical practice, i.e. registering, marketing and selling drugs. This may imply that research findings from IITs make it less often into practice, but this hypothesis has still not been conclusively verified. To the best of our knowledge, no prospective assessments of the impact of IITs on medical practice in terms of the utilization of research results through inclusion in systematic reviews and clinical guidelines have yet been made. While others have adopted a retrospective approach starting at the guideline and determining common characteristics of cited trials [37], we investigate and compare the impact of IITs and ISTs in a unique, prospective manner. The main purpose of this project was to assess whether there are differences in impact on clinical practice between IITs and ISTs, and between trials conducted in or outside Germany, i.e. primarily at German study sites or solely at study sites located outside Germany (2 × 2 factorial design). For that purpose, we determined and compared the proportion of clinical trials that have been published in a peer-reviewed journal as well as the inclusion of the publications (i.e. trials results) in secondary research articles like systematic reviews and clinical guidelines. We also analyzed, whether pre-selected study characteristics are associated with research impact. In this article we describe the rationale and design of this project and present the characteristics of the trials included in our study cohort.

### Objectives

**Fig. 1:**
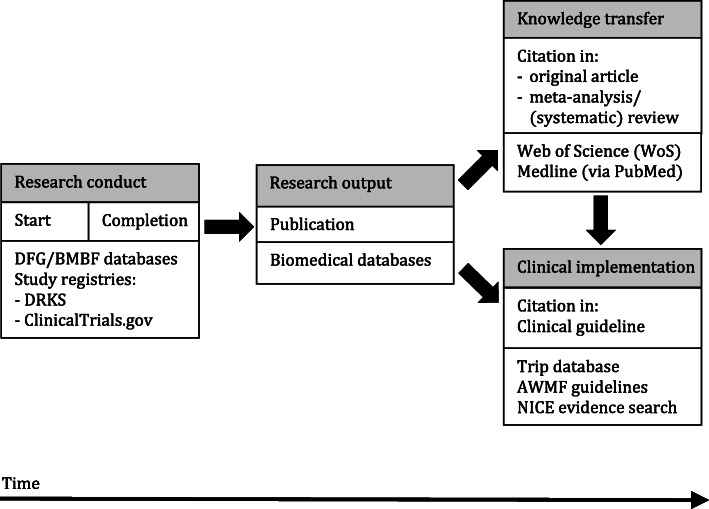
Research Impact Assessment

The main objective of this project is to evaluate the research impact of IITs and ISTs conducted in and outside Germany on clinical practice. For the assessment of research impact we followed the concepts of the earlier described Becker Medical Library Model for Assessment of Research Impact [34] (Fig. 1) and measured research impact on the basis of:

1. Publication proportion: proportion of trials with published study information (primary outcome),
2. Time from study completion to publication,
3. Visibility, i.e. findability of trial publication, measured as proportion of articles available and appropriately indexed in biomedical databases (e. g. Medline via PubMed),
4. Accessibility of publications measured as proportion of openly accessible publications (publication rights, e. g. open or closed access),
5. For German trials, broadness, i.e. internationality of the target group of the publication, measured as publication language (English or another language),
6. Impact of trial results on secondary research, measured as citation of publications by systematic reviews or meta-analyses,
7. Impact of trial results on clinical practice, measured as proportion of trials cited by clinical practice guidelines, either via the primary or via a secondary publication.

We also aimed to identify study characteristics associated with the impact of trials, e. g. sponsoring/funding of trials, study phase, i.e. phase of drug trials (I, II, II-III, III, IV) and non-drug trials, medical field, sample size, and type of intervention.

A secondary aim of the current project is to develop an innovative research tool based on the described “rationale and study design” to allow (semi-) automatic replications of future research impact analyses and/or for equivalent research tasks. This will help to gain insight into the dissemination of research knowledge and its impact over time.

## Methods

### Study cohort

Overall, the study cohort of our project comprises the following sub-cohorts:

- Public Germany (IITs with German contribution)
  - Public Germany gov (reference sub-cohort. IITs funded by the governmental organizations DFG and BMBF within their Clinical trials program)
  - Public Germany other (IITs funded by other non-commercial organizations or funding programs)
- Public International (IITs without German contribution)
- Commercial Germany (ISTs with German contribution)
- Commercial International (ISTs without German contribution)

#### Establishing the study cohort

In Germany, there are two main research funding organizations facilitating IITs within specific clinical trials funding programs since 2005, the German Research Foundation (DFG) [38] (also funding this project) and the German Federal Ministry of Education and Research (BMBF) [39]. IITs funded within these funding programs served as reference sub-cohort relating to the study characteristics for the creation of the comparison sub-cohorts. Between 2005 and the cut-off date of 31 Dec 2016, a total of 77 completed IITs were recorded and available in the databases of DFG and BMBF. For our research project, we focused on 60 trials (27 funded by the DFG and 33 by the BMBF) that met the following criteria:

- Therapeutic randomized controlled trial
- Interventional
- Multicenter
- Confirmatory
- Year of study application or study start: 2005 or later
- Study completion up to the cut-off date 31 Dec 2016

These characteristics were used as eligibility criteria for the creation of the comparison sub-cohorts.

Furthermore, we aimed to create sub-cohorts that did not differ substantially from each other concerning the sample size. Therefore, we limited the trials of the other sub-cohorts to the maximum number of participants of the reference sub-cohort, which was 4005.

The study information was taken from the funder websites and study registries.

### Creation of the sub-cohorts

To achieve a sufficient sample size of completed IITs with at least one study site in Germany, we complemented the 60 trials (Public Germany gov) retrieved from the DFG database German Project Information System (GEPRIS) [38] and the BMBF website [39] by an equal number of IITs funded by other German non-commercial organizations (Public Germany other) to a total of 120 (Public Germany) (Table 1).

**Table 1:** Study cohort. For search strategies, please refer to Additional file 1

	Sub-cohort	Source	Number of trials
IITs	Public Germany		120
	Public Germany gov	DFG/GEPRIS (*n* = 27), BMBF website (*n* = 33)	60
	Public Germany other	DRKS (*n* = 47), ClinicalTrials.gov (*n* = 13)	60
	Public International	ClinicalTrials.gov	200
ISTs	Commercial Germany^a^	DRKS (*n* = 42), ClinicalTrials.gov (*n* = 158)	171
	Commercial International	ClinicalTrials.gov	200

^a^ Due to an insufficient number of non-drug ISTs in the registries searched, we could only include 171 trials in the sub-cohort Commercial Germany (please refer to section “Balancing process” and Table 3)

The German Clinical Trials Register (DRKS) is an approved Primary Register in the WHO Registry network [40] and the central portal for information on clinical research in Germany [41]. It provides a complete and up-to-date overview of trials conducted in Germany. Therefore, we used the DRKS as the basis source for the German sub-cohorts Public Germany and Commercial Germany. We considered all eligible trials that were included in the DRKS and supplemented both sub-cohorts by trials drawn from ClinicalTrials.gov, a study registry including clinical trials conducted all over the world (210 countries) [42].

The trials for the two international sub-cohorts without German contribution (Public International and Commercial International) were all taken from ClinicalTrials.gov. For both sub-cohorts we included 200 trials each (please refer to “Sample size and statistical analysis”).

### Balancing of the sub-cohorts regarding study phase and study site location

Our study cohort is not a random sample of a defined population of studies but rather a compilation of sub-cohorts that are similar to the reference sub-cohort Public Germany with respect to important characteristics. Therefore, we decided to take into account the following study characteristics that are probably associated with the impact measures considered, by design: study phase and proportion of German study sites. We preferred to control for these characteristics by balanced design (also referred to as frequency matching) and not only by analysis.

The development process of a new drug normally goes through four study phases (Table 2). After passing phase 3, the drug is usually approved by a regulatory authority and, if successful, can then be used for health care of the general population. Phase 4 post-approval studies can follow. Therefore, it is evident that the probability for drug trials having an impact on medical practice changes with the study phase of the trial.

**Table 2:** Study phase classification scheme for drug trials and non-drug trials

Phase of drug trial/non-drug trial	Classification criteria
1/S	**Safety study** Question: “Is the therapy safe?” The trial focuses on the safety of a drug/therapy. The aim is to determine a safe dose range as well as the most common and serious adverse events associated with the drug/therapy. It is conducted with a small number of healthy participants.
2/A	**Pilot, feasibility, tolerability study** Question: “Is there a therapy effect?” The trial is explicitly defined as a pilot study or feasibility study or it can be assumed from the description that the therapy is either new or has never been investigated with regard to a specific outcome. The clinical trial collects initial data on drug/ treatment efficacy, i.e. whether or not a drug/treatment works in a specific study population, while continuing to monitor drug safety as well as short-term adverse events.
3/B	**Efficacy study** Question: “How large is the therapy effect?” or “Is the effect larger than the effect of other therapies?” Investigation and comparison of efficacy and safety under controlled conditions. The drug/therapy has already been tested, but more information is needed to establish the therapy. The clinical trial delves deeper into the safety and efficacy of a drug/treatment using different study populations, drug/treatment dosages, and combinations with other established drugs/treatments.
4/C	**Effectiveness study** Question: How can the effect be improved? Effectiveness and safety under real-life condition. The drug/therapy is approved for marketing/established, but needs to be optimized, implemented in practice and evaluated over a longer time period under routine conditions. Additional information on the safety, efficacy and/or optimal use of a drug/therapy is collected.

To prevent bias possibly occurring from systematic differences in study phase between the sub-cohorts, we balanced the three sub-cohorts Public International, Commercial Germany and Commercial International on the basis of the proportion of the specific study phase for both drug trials and non-drug trials (Table 2). Little is known about the influence of the study site location on research impact. Most (77%) of IITs included in the sub-cohort Public Germany were national trials, i.e. all participating study sites are located in Germany, but some of the trials (23%) have one or more study sites that are located outside Germany. To address this possibly biasing factor, we balanced the other comparison sub-cohort with German contribution, Commercial Germany, for this factor, i.e. the proportion of German study sites on all study sites.

### Balancing process

For each of the comparison sub-cohorts Commercial Germany, Public International and Commercial International, we selected all trials fulfilling the eligibility criteria from the trials registries and downloaded them into an Excel-database. The search strategies used to identify the trials in the registries are shown in the supplemental material for each sub-cohort (Additional file 1).

For each trial studying a drug or biological product, we determined the study phase according to the U.S. National Library of Medicine [43] classification scheme (phase 1–4). If reported, we verified and considered the study phase information as stated in the study registries, if not reported, we determined, according to the classification scheme, the study phase by ourselves on the basis of the information available in the registries (Table 2).

For non-drugs trials, a similar classification scheme is not commonly used. To be able to consider the development and implementation phase also for those non-drug interventions, we applied the same classification criteria as for drug trials and classified them as S, A, B, or C trials (Table 2).

For all trials of German ISTs (Commercial Germany), we calculated the proportion of German study sites.

To obtain comparable sub-cohorts, we used a stratified randomization. For each sub-cohort, we sorted both drug trials and non-drug trials by study phase. For the German ISTs, we used the proportion of German study site as a secondary sorting parameter within each study phase. All trials of the same study phase (for German ISTs also of the same study site proportion) were then numbered consecutively. On the basis of the percentages of study phase (and study site proportion for German ISTs) deriving from the sub-cohort Public Germany, we calculated the number of trials needed for each study phase and study site proportion for the comparison sub-cohorts. Then, for each sub-cohort, we selected the numbers of trials required for each study phase/study site proportion by using a random number generator. Duplicates were excluded and new trials re-randomized. Due to an insufficient number of non-drug ISTs in the registries, we considered all 78 identified eligible non-drug trials for inclusion in the sub-cohort Commercial Germany (Tables 1 and 3).

**Table 3:** Characteristics of included trials

Characteristics	IIT Public Germany gov No. of trials (%)	IIT Public Germany other No. of trials (%)	IIT Public Germany No. of trials (%)	IIT Public International No. of trials (%)	*IST Commercial Germany No. of trials (%)	IST Commercial International No. of trials (%)	Total No. of trials (%)
**Total**	60	60	120 (100)	200 (100)	171 (100)	200 (100)	691 (100)
**Registered in**^**a**^							
ClinicalTrials.gov	32 (53)	16 (27)	48 (40)	200 (100)	158 (92)	200 (100)	606 (88)
DRKS^b^	14 (23)	48 (80)	62 (52)	–	19 (11)	–	81 (12)
ISRCTN^c^	27 (45)	5 (8)	32 (27)	3 (1)	–	–	35 (5)
EudraCT^d^	40 (67)	10 (17)	50 (42)	18 (9)	88 (52)	33 (17)	189 (27)
**Study status**							
Completed	43 (72)	59 (98)	102 (85)	200 (100)	170 (100)	200 (100)	672 (97)
Prematurely ended	12 (20)	1 (2)	13 (11)		1 (< 1)		14 (2)
Still ongoing^e^	5 (8)	–	5 (4)				5 (< 1)
**Collaboration**							
International	19 (32)	7 (12)	26 (22)	44 (22)	71 (42)	69 (35)	210 (30)
National	40 (66)	53 (88)	93 (78)	156 (78)	100 (58)	131 (65)	479 (69)
Unclear	1 (2)	–	1 (< 1)	–	–	–	2 (< 1)
**Study size (Median = 150)**							
> 150	46 (76)	28 (47)	74 (62)	81 (40)	74 (43)	115 (58)	344 (50)
≤ 150	13 (22)	32 (53)	45 (38)	119 (60)	97 (57)	85 (42)	346 (50)
Unclear	1 (2)	–	1 (< 1)	–	–	–	1 (< 1)
**Number of primary outcome(s)**							
0	–	–	–	–	1 (1)	–	1 (< 1)
1	44 (73)	44 (73)	88 (73)	152 (76)	122 (71)	133 (67)	495 (72)
> 1 (range 2–36)	16 (27)	16 (27)	32 (27)	48 (24)	48 (28)	67 (33)	195 (28)
**Study phase drug trials**^**f**^							
Total	41 (68)	15 (25)	56 (47)	93 (47)	93 (54)	93 (47)	335 (48)
2	9 (15)	5 (8)	14 (12)	23 (12)	23 (13)	23 (12)	83 (12)
3	20 (33)	7 (12)	27 (22)	45 (23)	45 (26)	45 (23)	162 (23)
4	12 (20)	3 (5)	15 (13)	25 (13)	25 (15)	25 (13)	90 (13)
**Study phase non-drug trials**^**g**^							
Total	19 (32)	45 (75)	64 (53)	107 (53)	78 (46)	107 (53)	356 (52)
A	–	9 (15)	9 (7)	15 (7)	11 (7)	15 (7)	50 (7)
B	16 (27)	33 (55)	49 (41)	82 (41)	43 (25)	82 (41)	256 (37)
C	3 (5)	3 (5)	6 (5)	10 (5)	24 (14)	10 (5)	50 (7)

^a^ Several trials were registered in more than one trials registry, i.e. numbers do not sum up to the total numbers (100%)

^b^ DRKS: German Clinical Trials Register

^c^ ISRCTN: International Standard Randomized Controlled Trials Number registry

^d^ EudraCT: European Union Drug Regulating Authorities Clinical Trials Database

^e^ Status as of 24 April 2020

^f^ 15 drug trials of phase 2–3 were counted as phase 2; 24 non-drug trials of phase A-B were counted as phase A

^g^ In the sub-cohort “Commercial Germany”, we included all non-drug trials available in the study registries, resulting in slightly differing distributions of study phases among the 4 sub-cohorts

#### Data extraction

### Study characteristics extracted

For each included trial, we determined or extracted the following pre-defined study characteristics from the trials registries:

- Study title and acronym
- Start date of study (enrollment)
- Date of study completion
- Type of intervention (drug, surgery/procedure/medical device/manual therapy, behavioral, or other [e.g. biological agents, bone marrow cells, etc.])
- Medical field (according to the slightly modified version of the medical fields specified in the “(Model) Specialty Training Regulations 2003” of the German Medical Association [44])
- Number of participants (sample size)
- Number of primary outcomes
- Sponsor/Funding sources (commercial/non-commercial)
- Results reported in study register (yes/no)
- Publication references reported/linked to study register (yes/no)
- Other/secondary study register ID numbers, e.g. Eudra-CT [45], ISRCTN [46]

For trials with missing trial characteristics in DRKS or ClinicalTrials.gov, we also considered information reported in secondary study registries. For trials included in the Public Germany gov sub-cohort we also considered the basic study information available in the DFG and BMBF databases.

For further information on extracted study characteristics, please refer to Additional file 2.

### Piloting of the data extraction process

A manual describing the definitions for the data to be extracted was developed, i.e. for each variable it was described which data have to be extracted and how. According to these detailed data extraction instructions, the research team (AB, AI, KW, LR, SB, SL) independently double-extracted study data into the project database (MS Access 2010). The researchers were trained and data extraction was piloted on a test data set of 30 trials for which all researchers performed data extraction independently. We compared the results and discussed, edited as well as complemented the instructions, if and where necessary, before proceeding with the actual data extraction. Any discrepancies or disagreements were resolved through discussion or by consulting a third researcher until consensus was reached.

#### Assessing research impact

We examined research impact by assessing the proportion of trials that were published as well as the citation rate of their publication(s). In particular, we were interested in the proportion of trials and publications, respectively, cited by a systematic review or meta-analysis or a clinical guideline (Fig. 1).

Research translation from trial results to clinical implementation over time. The figure is based on the research impact assessment concepts of Sarli et al. [34] and was adapted for this project.

### Identifying primary research articles

For each included trial, we searched for corresponding articles included in biomedical databases to assess the proportion of conducted research that has been published.

#### Citations in registries

We examined whether a publication or its reference is directly attached or linked to the registry entry and whether trial results are reported in the study register.

#### Publications in bibliographic databases

Based on extracted data and keywords derived from the trials, we systematically searched in the following electronic databases for publications that correspond to the included trials:

- Study registries (DRKS, ClinicalTrials.gov, ISRCTN, EU Clinical Trials Register)
- Medline (via PubMed) [47]
- Cochrane Central Register of Controlled Trials (CENTRAL) [48]
- LIVIVO (interdisciplinary search engine for life sciences literature) [49]
- Web of Science (WoS) [50]
- Google scholar [51]
- Google [52]
- Study website
- PubMed tools “Similar articles “and “Cited by“

For each trial, the search was conducted in the following order and with the following search terms: 1. Register Identifier (NCT ID, DRKS ID, etc.^1^); 2. Acronym; 3. Name of applicant/investigator(s); 4. Study title; 5. Study methods/PICO (Population, Intervention, Comparison, Outcome) components [53]; 6. Funding number.

References of publications that corresponded to the trial were downloaded into a reference management database (endnote). The full text of the article was retrieved, e.g. by the departmental librarian, and attached to the corresponding reference. If we were unable to decide on the eligibility of an article based on the database entry, we also retrieved the full text article for further evaluation and decision. We only considered full publications, i.e. articles that contain at least some information on the study’s objectives, methods and/or results that were published in a scientific peer-reviewed journal

### Identifying secondary research articles

#### Cited by reviews

We downloaded the bibliographic citations of all references, including the digital objective identifier (DOI), citing the publication from the databases Medline (via PubMed) [47] and WoS [50] by means of the “Cited by” function (PubMed/Medline) and the “Times cited” function (WoS). This was done automatically by a program developed by one of the authors (KN). To determine which of the articles citing the publication is a systematic review or meta-analysis, we used Epistemonikos, a multi-collaborative database of health research evidence and the largest source of systematic reviews and other types of scientific evidence [54]. Its primary aim is to identify all systematic reviews relevant for health-decision making by regularly screening multiple electronic databases and other sources, including Cochrane Database of Systematic Reviews (CDSR), PubMed, Excerpta Medica database (EMBASE), Cumulative Index to Nursing and Allied Health Literature (CINAHL), Psychological Information (PsycINFO) database, Latin American and Caribbean Health Sciences Literature (LILACS), the Campbell Collaboration Online Library, the Joanna Briggs Institute (JBI) Database of Systematic Reviews and Implementation Reports, and the Evidence for Policy and Practice Information and Co-ordinating Centre (EPPI-Centre) Evidence Library [55–62]. Epistemonikos classifies potentially eligible articles by a machine-learning algorithm and checked by the network of human collaborators. Apart from systematic reviews, Epistemonikos does also include broad syntheses, i.e. summaries of systematic reviews [63].

We consider comparing the citing references with the content of Epistemonikos a reliable method to determine the publication type and also deem it suitable for publications that are not indexed with a publication type, e.g. because they are not included in Medline.

We matched the DOI of each downloaded citing reference with the record-DOIs included in Epistemonikos. For publications without DOI, we matched the publication title. For this purpose, a master list of all records was provided by Epistemonikos on request (as of 28 June 2019), containing the bibliographic citation information of the reference DOI, journal title, publication year, PubMed identifier (PMID)/Cochrane ID, and Epistemonikos’ ID and classification type (broad-synthesis or systematic review). The matching process was done automatically by a program written by one of our authors (KN) in Python programming language [64]. The references of all identified matching pairs was entered into the project Access database and linked to the reference of the “parent” publication.

For further assessment of the impact of the trial results in clinical guidelines we focused on the reviews identified by this process.

#### Cited by clinical guidelines

To identify clinical guidelines that include results deriving from our trial cohort, we manually searched the following three guideline databases: the search portal for German guidelines (AWMF Guidelines) and, for international guidelines, the Turning Research Into Practice (TRIP) database and National Institute for Health and Care Excellence (NICE) evidence search. The guideline database of the Association of the Scientific Medical Societies (AWMF) of Germany contains guidelines and related documents of all member medical specialist societies in Germany [65]. The Trip medical database [66] provides a search engine that enables healthcare professionals to easily search, find and use research evidence (e.g. international guidelines) in practice and/or care. NICE evidence search [67] offers free access to high quality evidence on (public) health, drugs and health technologies, social care, and healthcare management and implementation. It contains consolidated and synthesized evidence from various established sources such as the British National Formulary (BNF), Clinical Knowledge Summaries (CKS), Scottish Intercollegiate Guidelines Network (SIGN), the Cochrane Library, and Royal Colleges [68–71]. A variety of documents can be retrieved from NICE including systematic reviews, guidance, evidence summaries and patient information [72].

We searched for guidelines citing the original publication and/or the systematic review(s) identified by the matching process mentioned above. The search was performed by using (parts of) the article title, name of first author, intervention, and disease.

We also searched for the register identifier of the trials to identify guidelines citing study information or results included in the trial registers.

We complemented the manual search by an automatic search tool programmed by KN (please refer to “Methods”,” Sub-study”).

### Characteristics of primary research articles

The following information on the publication characteristics of an original article was extracted:

- Reference information (author, title, journal, volume, issue, pages)
- Type of publication: protocols, method papers, or result articles
- Date of publication (electronic version)
- Date of publication (print version)
- DOI
- Type of research article
- Country of first author
- Free full-text article availability (open/closed access)
- Free PubMed Central (PMC) article availability (yes/no)
- Distribution rights (creative commons license)
- Search term(s) by which publication was found
- Database(s) where publication was found
- Study registry identifier as reported in database and/or article
- Language of article

### Characteristics of secondary research articles

#### Systematic reviews and meta-analyses

We determined and extracted the following characteristics of secondary research articles:

- Reference information (author, title, journal, volume, issue, pages)
- Date of publication (electronic version)
- Date of publication (print version)
- DOI
- Type of review according to Epistemonikos classification: systematic review or broad synthesis
- Context of publication citation: whether the publication is cited in general, e.g. in the introduction or discussion section, or study results are included or excluded in the systematic review or meta-analysis

#### Guidelines

For the retrieved guidelines we extracted the following characteristics:

- Title
- Year of publication
- Guideline identifier (e. g. AWMF register number)
- Database in which the guideline was found: TRIP, AWMF or NICE
- Language of guideline: English, non-English (e. g. German, French, etc.)
- Guideline quality: S1/S2/S3 (only applicable for German AWMF guidelines^2^)

#### Sample size and statistical analysis

With the size of the sub-cohort Public Germany being restricted to *n* = 120 trials, it is possible to estimate the proportion of published trials (primary outcome) with a standard error (SE) of less than 0.05 in this sub-cohort. The intended sample sizes of *n* = 200 trials for the other three sub-cohorts will lead to SEs of about 0.035 for the corresponding estimated proportions in these sub-cohorts. Since the comparison of sub-cohorts with regard to publication proportions will be based on the more informative outcome time to publication, these sample sizes were chosen to achieve a power of over 90% (significance level of 5%) for a hazard ratio of 1.6 (increase of publication hazard) or 0.625 (decrease of publication hazard) assuming an overall publication proportion of 50% over a long follow-up period. There will be no adjustments for the number of comparisons. The time to publication analysis will properly take different follow-up lengths for the individual studies into account. In our planned analysis, we will present Kaplan-Meier plots of time-to-publication for the four sub-cohorts as well as results of Cox regression analyses, considering study characteristics. The intended sample sizes for the study cohorts will provide reasonable power for the detection of moderate to large differences between IITs and ISTs, also for the other endpoints considered.

Although all trials included in the sub-cohorts met the inclusion criteria and were balanced for the study phase, and the German IITs and ISTs for the proportion of German study sites, it might be possible that the sub-cohorts are still heterogeneous for other factors. This makes a comparison of the research impact susceptible to bias. Therefore, we attempted to create comparable groups by: a) pre-defining inclusion criteria, and b) conducting a propensity score analysis to evaluate additional influencing factors [73–75]. Study characteristics that turn out to have an influence on research impact will be adjusted for in the regression model to address confounding. In addition to the regression analyses, we planned a propensity score analysis as a form of sensitive analysis, where we use documented study characteristics that are not controlled for by design. These are, for instance, study status, study size, and number of primary outcomes. With this approach we are able to minimize possible bias when assessing the real effect of research impact.

Values will be quantified by means of absolute number, percentage, median and range.

#### Sub-study: developing and validating a robust semi-automatic tool for follow-up

We also developed and validated a robust methodological tool that allows following-up trials and periodically replicating research impact analyses over time in a semi-automated manner. The tool*,* called *DOIScout*, comprises two main features. The first main feature is an automatic search for publications using their study register identifier (e. g. NCT01234567). The second main feature focuses on the impact of the identified publications using the PubMed and WoS citation tracking function, i.e. how many times a publication has been cited by other articles (PubMed function “Cited by”, WoS function “Times Cited”). Moreover, the tool is also designed to automatically search specific guideline databases (AWMF, TRIP, NICE) for guidelines citing the publication. The DOIScout collects the bibliographic information of the identified citations and the sources (databases) from where they were found. The tool also includes several secondary features aiming at facilitating workflows, for example importing PubMed- and WoS-files and downloading full text articles (PDFs) when available. Ultimately, the DOIScout will be made available as an open-source and user-friendly tool. Thus, it can be used for related research projects so that the scientific work and the scientific community can benefit from this tool.

## Results

### Characteristics of included trials

#### Total

Our final study cohort included a total of 691 trials (Table 3).

#### Registered in

We also extracted study IDs of other/secondary study registries reported in DRKS or ClinicalTrials.gov. We identified IDs from two other trial registries: The ISRCTN (originally stood for International Standard Randomised Controlled Trial Number) registry which includes RCTs and other types of interventional trials as well as observational trials assessing the efficacy of health interventions in humans [46]. The other registry, the European Union Clinical Trials Register, is a register where protocol and results information on clinical trials included in the European Union Drug Regulating Authorities Clinical Trials (EudraCT) database, the European Clinical Trials Database for clinical trials testing medicinal products, are made publicly available [45].

One third of our trials (224; 32%) were included in these two secondary study registries, 5% in the ISRCTN registry and 27% in EudraCT.

In order to be registered, at least one site has to be located within the European Union. In Germany, a planned clinical drug trial must be registered in EudraCT before an application for approval of the trial can be submitted. This means that all 149 drug-trials of our German sub-cohorts should be included in EudraCT and we found almost all: 50 of 56 (89%) of the trials included in Public Germany and 88 of 93 (95%) of Commercial Germany trials.

#### Study status

Even though the search strategies were designed to only identify completed trials, information from registries and other sources revealed that 19 out of 691 (3%) trials were not completed according to protocol: Fourteen (2%) were closed but ended prematurely, five trials (< 1%), all belonging to the sub-cohort Public Germany gov, were still ongoing at the time of data extraction. The reason for this was that in the source, from which the trials derived, studies were labelled as completed when the funding period had elapsed, irrespective of the actual completion date.

#### Collaboration

We also determined the collaboration of a study, i.e. whether study sites in one or more countries participated in the trial. Most of the trials (69%) included in our study cohort were national trials, i.e. they were conducted in one country, 30% were conducted in more than one country. This difference was more prominent in IITs (78% versus 22%) than in ISTs (Table 3). The number and proportion of national/international trials were identical between the sub-cohorts Public Germany and Public International, because we balanced for these criteria, i.e. proportion of German study sites among all study sites.

#### Study size

The median sample size of all included trials was 150. Of the sub-cohorts Public Germany and Commercial International, a higher proportion of trials had a sample size > 150 than of the sub-cohorts Public International and Commercial Germany trials.

#### Number of primary outcomes

In all sub-cohorts, 525 (76%) trials had one pre-defined primary endpoint, but for 30 of those, more than one time of measurement was stated, resulting in 495 (72%) studies with one specific primary endpoint measured at one specific time point. Overall, 28% of the studies had more than one primary outcome(s), the maximum number was 36.

#### Study phase

We balanced the comparison sub-cohorts for the study phase on the basis of our reference sub-cohort Public Germany. None of the trials included in sub-cohort Public Germany belonged to phase 1 or S, so that we did not include any of those trials in our study cohort (please refer to “Methods” and Tables 1 and 3). About half (27 of 56; 48%) of the drug trials belonged to phase 3, 25% to phase 2 and 27% to phase 4. For non-drug trials, even more (49 of 64; 77%) belonged to the corresponding study development phase B, 14% were phase A trials and 9% phase C.

According to the distribution in the reference sub-cohort Public Germany, we aimed to include 47% drug trials and 53% non-drug trials in each of the comparison sub-cohorts. For the sub-cohort Commercial Germany, not enough non-drug trials (only 78 instead of 107) could be identified in the study registries. This reduced the total number of included trials for this sub-cohort and led to a difference in the proportion of non-drug trials versus the drug trials as well as to a difference in the study phase proportions. Please refer to Table 3 for detailed characteristics of all included trials.

## Discussion

In this present project we assess and compare the research impact of investigator initiated trials and industry sponsored trials conducted in Germany and internationally from all medical fields in a unique prospective manner. Starting our investigation at the very first beginning of a study, the stage of funding application and registration, we follow the study’s pathway up to its impact and perception in clinical practice by assessing its inclusion in systematic reviews and/or guidelines.

### Strengths and limitations

A strength of our research project is the special focus put on clinical implementation indicators to effectively assess research impact on clinical practice. Inclusion in systematic reviews and clinical guidelines is such an indicator to measure the use of research findings in medical practice. We are also able to make more accurate assessments of research impact by not only examining whether retrieved publications were cited and used in systematic reviews but also *how* they were used, i.e. included, excluded or used otherwise. This is of crucial importance as the inclusion of study results and not only their citation in systematic reviews is the critical factor that indicates the contribution of study results to the body of evidence. We recorded and analyzed the reasons for non-inclusion of original articles in systematic reviews. Thereby we may gain a better understanding of the trials involved in the development of clinical or practice guidelines and in decision-making processes.

Another strength of our project lies in the development of a research tool to semi-automatically replicate and update the analyses over time. Currently unpublished trials can be followed up in later impact assessments. Finally, our trial cohort comprises trials of a broad range of medical fields so that our results are comprehensively valid.

Since we included trials from a specific predefined time period (2005–2016), not sufficient time may have passed for some of the trials to publish the results and to be included in systematic reviews or clinical guidelines. This applies especially to the trials that were completed at the end of that period. We intend to estimate the effect by a time-to-publication analysis.

For trials that were completed early in that time period, i.e. with sufficient time to be published, the publication proportion over time can be calculated. These values allow predicting the proportion of “missing” publications, systematic reviews and clinical guidelines that could not be included in our analysis, because insufficient time had elapsed, but will probably be published and may have an impact at a later time. This limitation is addressed within this project by the development of the DOIScout, which allows replicating and updating the analyses.

Minor limitations derived from incomplete or outdated trial information in trials registries and the limited availability of trials in the sub-cohort Commercial Germany.

Although the search strategy strictly aimed at only identifying completed trials, five trials (< 1%) were still ongoing (please refer to “Results” and Table 3).

For the sub-cohort Commercial Germany, not enough non-drug trials were included in the registries so that we considered all trials that were available, regardless of their study phase. Therefore, the balancing criteria were only partially fulfilled for this sub-cohort; this will be considered in the analysis (please refer to “Methods” and Table 3).

Information on funding source and involvement (planning or conduct) of commercial organizations in the study was not reported for most of the trials in the registries. Therefore, we could not compare our sub-cohorts for these study characteristics.

### Comparison with similar trials

In the scientific literature different attempts exist to “measure” and analyze the impact of clinical studies on medical practice and to identify underlying factors that might have an influencing effect. A systematic review provided an overview of 24 methodological frameworks that had been identified to measure research impact in health care [76]. The frameworks described varied concerning development process and impact categories. Overall, with respect to the time to impact (‘short-term’, ‘mid-term’, or ‘long-term’) and across the 24 included methodological frameworks, five major categories were proposed: (1) primary research-related impact, (2) influence on policy making, (3) health and health systems impact, (4) health-related and societal impact, and (5) broader economic impact, and 80 different metrics to measure research impact.

This systematic review also includes the Becker Medical Library Model for Assessment of Research used within the current project [34]. In a theoretical approach, the authors showed clear pathways of diffusions for results of a research study, categorized as research output, knowledge transfer, clinical implementation, and community benefit.

In these pathways, citation analysis is one metric of impact that is frequently used in research.

In our project, we focused on citation analysis, but in a novel approach: we followed the life cycle of trials prospectively, i.e. from the beginning, the registration, up to the publication of results in primary scientific publications and a possible inclusion in reviews and guidelines. In this manner we aimed to gather not only information about “successful” trials with citations and impact but also about the “losses” during that lifecycle. Thus, we were able to identify trials that remained unpublished and/or had no impact. This allowed collecting quantitative data about those “losses” and identifying possible explanatory reasons and factors.

Bibliometric citation analysis can be performed and used in different ways. In a brief comparison of the types of citation analysis commonly used in literature, we will discuss their strengths and limitations below and show what our approach can add to the existing knowledge.

A common tool to assess the impact of a study is to simply count how often a publication has been cited. This prospective approach is frequently used, for example, to determine the most “successful” articles and authors in the various medical fields. Annually, various articles about “The 100 most cited manuscripts/articles” in specific medical field are published [77–80]. The data for these analyses can be easily obtained via bibliometric databases, such as Web of Science and Medline (PubMed), making this approach a quick and easy way to identify publications that are highly perceived by the scientific community. Furthermore, database providers themselves provide search tools based on citation analysis and release annual lists with the world’s most highly cited researchers, i.e. those who produced papers ranking in the top 1% by citations for their field [81]. However, this ranking of publications and authors does not consider the content of the article and does not give information about its real value for medical practice.

Another type of citation analysis is a retrospective approach, i.e. starting at review or guideline level and analyzing the references that were cited in there. An example for this approach is the study published by Pallari et al. [82], in which the authors assessed the impact of cited research evidence underpinning the development of cancer clinical practice guidelines (CPGs) by the professional bodies of the European Society for Medical Oncology (ESMO), NICE and the Scottish Intercollegiate Guideline Network (SIGN). For this purpose they collected 101 cancer CPGs from the websites of ESMO [83], NICE and SIGN and analyzed their cited references. They found heterogeneity in the cancer CPGs of ESMO, NICE and SIGN, which they explained by the heterogeneity in the evidence base used for the development of these CPGs.

Similarly, a study by Kryl et al. [37] assessed the feasibility of using research papers cited in clinical guidelines to track the influence of particular funding sources. They analyzed authorship and funding attribution of research cited in two NICE clinical guidelines of two medical specialties. Key findings of the study included the potential of citation analysis in clinical guidelines as a tool for evaluating research impact, in particular for investigating links between funding sources and possible changes in clinical practices as a result of guideline use.

Such retrospective analyses can give important information on specific characteristics of guidelines, i.e. topicality and types of research included, but are not sufficient and appropriate to assess clinical research impact comprehensively [84, 85]. Further insights gained from cited publications, e.g. in reviews or original articles, are limited as long as the manner of use of the study results is not taken into account. Furthermore, statement can only be made about the “successful” trials, i.e. those that have been included in the guideline.

The important question about the “losses” concerning clinical research impact and the underlying reasons can only be addressed by evaluating and comparing that group of trials and corresponding publications that were not cited.

In our project we followed up a pre-defined trial cohort in time by using the prospective citation analysis. With this approach, we were able to investigate the fate of all trials, i.e. which of the trials were published and/or included in other research articles or not, and for what reason.

With our quantitative collected dataset and prospective approach we can answer the following important questions: What is the proportion of clinical trials that do not have impact in reviews and guidelines? What are the possible reasons for this? Are their results not adequately published or findable? Have they been excluded and for what reasons?

## Conclusions

With the results of this proposed research project, we wish to deepen our understanding and add to the knowledge base of the impact assessment of biomedical research on clinical practice and healthcare policy. Biomedical research is highly resource consuming (time, personnel, finances, etc.), involving multiple stakeholders such as researchers, clinicians and patients.

The current project may not only add important insights and arguments for the strategic and efficient allocation of scarce research resources, but could also facilitate providing accountability to the different stakeholders involved.

## Supplementary information

**Additional file 1.** Search Strategies: Search strategies as applied to the trial databases DRKS and ClinicalTrials.gov

**Additional file 2.** Study characteristics: Description of the study characteristics extracted

## Data Availability

The dataset(s) supporting the conclusions of this article is (are) included within the article (and its additional files).

## Abbreviations

AWMF: Arbeitsgemeinschaft der wissenschaftlichen medizinischen fachgesellschaften (German association of the scientific medical societies)
BMBF: Bundesministerium für bildung und forschung (Federal ministry of education and research)
CINAHL: Cumulative index to nursing and allied health literature
BNF: British national formulary
CENTRAL: Cochrane central register of controlled trials
CDSR: Cochrane database of systematic reviews
CPGs: Clinical practice guidelines
CKS: Clinical knowledge summaries
DFG: Deutsche forschungsgemeinschaft (German research foundation)
DOI: Digital object identifier
DRKS: Deutschen register klinischer studien (German clinical trials register)
Embase: Excerpta medica database
EPPI-Centre: Evidence for policy and practice information and co-ordinating centre
ESMO: European society for medical oncology
EudraCT: European union drug regulating authorities clinical trials database
GEPRIS: German project information system
IITs: Investigator initiated trials
ISRCTN: International standard randomized controlled trials number
ISTs: Industry sponsored-trials
JBI: Joanna briggs institute
LILACS: Latin American and caribbean health sciences literature
NCT: National clinical trial (number)
NICE: National institute for health and care excellence
PMC: PubMed central
PMID: PubMed (unique) identifier
PsycINFO: Psychological Information
RCT: Randomized controlled trial
SIGN: Scottish intercollegiate guidelines network
TRIP: Turning research into practice
WoS: Web of science

## Footnotes

1 Clinical trial identification number assigned by the study registry, e. g. ClinicalTrials.gov.

2 The AWMF S-classification scheme classifies guidelines into classes S1, S2 and S3. Class S1 guidelines consist of action recommendations by experts but lack a systematic development process. S2 guidelines are either developed using a systematic analysis of the scientific evidence (S2e) or a structured consensus finding by a representative body (S2k). S3 guidelines combine both aspects and form the highest class of guidelines.
