# Supplementary material for: Impact of investigator initiated trials and industry sponsored trials on medical practice (IMPACT): rationale and study design
Source: BMC Med Res Methodol. 2020 Oct 2;20:246. doi: 10.1186/s12874-020-01125-5 (PMC7532587; doi:10.1186/s12874-020-01125-5)
Supplement: Supplementary file 1 — Additional file 1. Search Strategies: Search strategies as applied to the trial databases DRKS and ClinicalTrials.gov [file 12874_2020_1125_MOESM1_ESM.docx]

**Additional file 1: Search strategies**

**Search strategy applied to select studies from the German Clinical Trials Register (DRKS):**

Investigator Sponsored/Initiated Trial (IST/IIT): any

Study type: interventional

Purpose: therapy

First registration: 01/01/2005

Setting of the remaining selection fields: any

**Search strategies applied to select studies for the comparison sub-cohorts from ClinicalTrials.gov:**

**Public Germany other:** At least one study site located in Germany and lead sponsor is not industry:

SEARCH[STUDY] ( NOEXPAND EXACT "Treatment" [DESIGN-PRIMARY-PURPOSE] AND NOEXPAND EXACT "Randomized" [DESIGN-ALLOCATION] AND NOEXPAND EXACT NOT ( NOTEXT OR "1" ) [NUMBER-OF-LOCATIONS] AND NOEXPAND ( NOTEXT OR ( "0" : "4005" ) ) [ENROLLMENT] AND NOEXPAND ( "January 2005" : "August 2020" ) [START-DATE] AND NOEXPAND EXACT "Completed" [OVERALL-STATUS] AND NOEXPAND EXACT "Germany" [COUNTRIES] AND NOEXPAND EXACT NOT "INDUSTRY" [LEAD-SPONSOR-CLASS] )

**Public International:** No study site located in Germany and lead sponsor is not industry:

SEARCH[STUDY] ( NOEXPAND EXACT "Treatment" [DESIGN-PRIMARY-PURPOSE] AND NOEXPAND EXACT "Randomized" [DESIGN-ALLOCATION] AND NOEXPAND EXACT NOT ( NOTEXT OR "1" ) [NUMBER-OF-LOCATIONS] AND NOEXPAND ( NOTEXT OR ( "0" : "4005" ) ) [ENROLLMENT] AND NOEXPAND ( "January 2005" : "August 2020" ) [START-DATE] AND NOEXPAND EXACT "Completed" [OVERALL-STATUS] AND NOEXPAND EXACT NOT "Germany" [COUNTRIES] AND NOEXPAND EXACT NOT "INDUSTRY" [LEAD-SPONSOR-CLASS] )

**Commercial Germany:** At least one study site located in Germany and lead sponsor is industry:

SEARCH[STUDY] ( NOEXPAND EXACT "Treatment" [DESIGN-PRIMARY-PURPOSE] AND NOEXPAND EXACT "Randomized" [DESIGN-ALLOCATION] AND NOEXPAND EXACT NOT ( NOTEXT OR "1" ) [NUMBER-OF-LOCATIONS] AND NOEXPAND ( NOTEXT OR ( "0" : "4005" ) ) [ENROLLMENT] AND NOEXPAND ( "January 2005" : "August 2020" ) [START-DATE] AND NOEXPAND EXACT "Completed" [OVERALL-STATUS] AND NOEXPAND EXACT "Germany" [COUNTRIES] AND NOEXPAND EXACT "INDUSTRY" [LEAD-SPONSOR-CLASS] )

**Commercial International:** No study site located in Germany and lead sponsor is industry:

SEARCH[STUDY] ( NOEXPAND EXACT "Treatment" [DESIGN-PRIMARY-PURPOSE] AND NOEXPAND EXACT "Randomized" [DESIGN-ALLOCATION] AND NOEXPAND EXACT NOT ( NOTEXT OR "1" ) [NUMBER-OF-LOCATIONS] AND NOEXPAND ( NOTEXT OR ( "0" : "4005" ) ) [ENROLLMENT] AND NOEXPAND ( "January 2005" : "August 2020" ) [START-DATE] AND NOEXPAND EXACT "Completed" [OVERALL-STATUS] AND NOEXPAND EXACT NOT "Germany" [COUNTRIES] AND NOEXPAND EXACT "INDUSTRY" [LEAD-SPONSOR-CLASS] )
