# Supplementary material for: Impact of investigator initiated trials and industry sponsored trials on medical practice (IMPACT): rationale and study design
Source: BMC Med Res Methodol. 2020 Oct 2;20:246. doi: 10.1186/s12874-020-01125-5 (PMC7532587; doi:10.1186/s12874-020-01125-5)
Supplement: Supplementary file 2 — Additional file 2. Study characteristics: Description of the study characteristics extracted [file 12874_2020_1125_MOESM2_ESM.docx]

**Additional file 2: Study characteristics**

| **Study characteristic** | **Description** |
| --- | --- |
| Title | Official title of the study |
| Acronym | Acronym of the study |
| Register ID | Study identifier of all identified study register entries |
| Start date | Date of start of enrollment/recruitment |
| Completion status | Completed according to protocol, prematurely ended, still ongoing, or unclear/no information |
| Completion date | Date of study completion or discontinuation |
| Discontinuation | Reasons for study discontinuation |
| Intervention type | Drug, surgery, medical device, behavioral |
| Control type | Type of control treatment (no treatment, active, placebo) |
| Study phase of drug trials | Phase I, phase II, phase III, phase IV, not applicable (for non-drug trials), unclear |
| Study phase of non-drug trials | Classification scheme see table 4 |
| Number of study arms | Number of study arms |
| Design | Parallel, cross-over, factorial |
| Participant age | Age of participants |
| Participant gender | Gender of participants |
| Sample size | Total number of study participants |
| Collaboration | International (study conducted in more than one country), national (study conducted on one country) |
| Study site countries | Countries of study sites participating in study |
| Study site number | Number of study sites in each country |
| Sponsor | Sponsor of study (industry/non-industry) |
| Funding | Funder of study (industry/non-industry) |
| Funding type | Full or partial funding |
| Industry involvement | Industry involvement in study planning and conduction (yes/no) |
| Medical field | Medical field, according to the “(Model) Specialty Training Regulations 2003” of the German Medical Association, in which study can be categorized, e.g. cardiology, psychiatry |
| Primary outcome number | Number of primary outcome(s) |
| Primary outcome type(s) | Patient relevant (e.g. survival, mortality, Quality of Life), surrogate (blood pressure, cholesterol), both |
| Study protocol availability | Availability of full study protocol via study register (yes/no) |
| Results in study register | Results available in registries (yes/no) |
